# Profiling of plasma biomarkers in the context of memory assessment in a tertiary memory clinic

**DOI:** 10.1038/s41398-023-02558-4

**Published:** 2023-07-25

**Authors:** Marco Bucci, Marina Bluma, Irina Savitcheva, Nicholas J. Ashton, Konstantinos Chiotis, Anna Matton, Miia Kivipelto, Guglielmo Di Molfetta, Kaj Blennow, Henrik Zetterberg, Agneta Nordberg

**Affiliations:** 1 Department of Neurobiology, Care Sciences and Society, Centre for Alzheimer Research, Division of Clinical Geriatrics, Karolinska Institutet, SE-14183 Stockholm, Sweden; 2 Theme Inflammation and Aging, Karolinska University Hospital, SE-14186 Stockholm, Sweden; 3 Medical Radiation Physics and Nuclear Medicine, Karolinska University, SE-14186 Stockholm, Sweden; 4 Department of Psychiatry and Neurochemistry, Institute of Neuroscience and Physiology, Sahlgrenska Academy, University of Gothenburg, SE-43180 Mölndal, Sweden; 5 Department of Neurology, Karolinska University Hospital, SE-14186 Stockholm, Sweden; 6 Clinical Neurochemistry Laboratory, Sahlgrenska University Hospital, SE-43180 Mölndal, Sweden; 7 Department of Neurodegenerative Disease, UCL Institute of Neurology, Queen Square, London, WC1N 3BG UK; 8 UK Dementia Research Institute at UCL, London, WC1N 3BG UK; 9 Hong Kong Center for Neurodegenerative Diseases, Clear Water Bay, Hong Kong, China; 10 Wisconsin Alzheimer’s Disease Research Center, University of Wisconsin School of Medicine and Public Health, University of Wisconsin-Madison, Madison, WI 53792 USA

**Keywords:** Diagnostic markers, Predictive markers

## Abstract

Plasma biomarkers have shown promising performance in research cohorts in discriminating between different stages of Alzheimer’s disease (AD). Studies in clinical populations are necessary to provide insights on the clinical utility of plasma biomarkers before their implementation in real-world settings. Here we investigated plasma biomarkers (glial fibrillary acidic protein (GFAP), tau phosphorylated at 181 and 231 (pTau181, pTau231), amyloid β (Aβ) 42/40 ratio, neurofilament light) in 126 patients (age = 65 ± 8) who were admitted to the Clinic for Cognitive Disorders, at Karolinska University Hospital. After extensive clinical assessment (including CSF analysis), patients were classified as: mild cognitive impairment (MCI) (*n* = 75), AD (*n* = 25), non-AD dementia (*n* = 16), no dementia (*n* = 9). To refine the diagnosis, patients were examined with [^18^F]flutemetamol PET (Aβ-PET). Aβ-PET images were visually rated for positivity/negativity and quantified in Centiloid. Accordingly, 68 Aβ+ and 54 Aβ– patients were identified. Plasma biomarkers were measured using single molecule arrays (SIMOA). Receiver-operated curve (ROC) analyses were performed to detect Aβ-PET+ using the different biomarkers. In the whole cohort, the Aβ-PET centiloid values correlated positively with plasma GFAP, pTau231, pTau181, and negatively with Aβ42/40 ratio. While in the whole MCI group, only GFAP was associated with Aβ PET centiloid. In ROC analyses, among the standalone biomarkers, GFAP showed the highest area under the curve discriminating Aβ+ and Aβ– compared to other plasma biomarkers. The combination of plasma biomarkers via regression was the most predictive of Aβ-PET, especially in the MCI group (prior to PET, *n* = 75) (sensitivity = 100%, specificity = 82%, negative predictive value = 100%). In our cohort of memory clinic patients (mainly MCI), the combination of plasma biomarkers was sensitive in ruling out Aβ-PET negative individuals, thus suggesting a potential role as rule-out tool in clinical practice.

## Introduction

In a clinical setting, molecular imaging and fluid biomarkers (i.e., CSF) have proven useful in diagnostic assessment of patients with memory complaints [1, 2]. Although some of the available biomarkers (i.e., Aβ PET, CSF) have early changepoints [3–5], their application is connected to high costs, limited accessibility of radioactive tracers, or invasiveness. In this context, plasma biomarkers represent an attractive alternative due to the cost-effectiveness of commercial assays, along with their low invasiveness, and the accessibility of blood-sampling procedures [6]. More importantly, early and accessible diagnostic tools for Alzheimer’s disease (AD) are a clinical research priority, since they could facilitate timely diagnosis, better social management, identification of patients suitable for amyloid lowering treatment or at risk of deterioration in disease management, and development of disease-modifying drugs.

In recent years, we have seen a great increase in studies on AD-associated plasma biomarkers. In research cohorts comprised of well-profiled cases, novel plasma biomarkers have shown good accuracy in differentiating between clinically-diagnosed AD, frontotemporal dementia (FTD), and Lewy body dementia (LBD) [7], as well as between amyloid-positive and amyloid-negative individuals [8]. Among the candidate biomarkers, the plasma Aβ42/40 ratio has often shown the highest value in predicting Aβ PET status as defined by PET [8–11], as well as conversion rates from mild cognitive impairment (MCI) to dementia [12]. On the other hand, studies showed that Aβ42/40’s ability to predict Aβ PET status is affected by the type of assay used to quantify Aβ species [13]. At the same time, elevated levels of plasma glial fibrillary acidic protein (GFAP) were reported in elderly individuals at high risk of AD (cognitive normal older individuals with low plasma Aβ42/40 ratio) [14], as well as in carriers of deterministic autosomal dominant AD mutations a decade prior to expected symptomatic disease manifestation [15]. In autopsy studies, plasma pTau181 and pTau231 had the highest sensitivity and specificity in detecting AD neuropathological changes compared to pathology diagnoses and ratings [16].

Notably, research cohorts tend to have strict inclusion and exclusion criteria, which lead to a higher degree of patient homogeneity, facilitating the interpretability of results. In clinical settings, however, more patients tend to fall outside these stringent frameworks in both demographic and clinical characteristics (i.e., disease subtypes, comorbid pathologies), which might affect the magnitude of difference between diagnostic groups. Therefore, studies in clinical populations should provide valuable insights on the clinical utility of plasma biomarkers ahead of their incorporation in a real-world setting.

Taken together, these considerations motivate the present study, where we aim to assess the value of the promising plasma biomarkers in a cohort of tertiary clinic patients with cognitive complaints, including patients with different diagnosis ranging from prodromal to dementia stages of AD and other non-AD dementia disorders. The plasma biomarkers assessed such as Aβ42/40, pTau231, pTau181, neurofilament light (NfL), and GFAP might be differentially associated with various pathological events observed in AD, such as amyloidosis, tauopathy, neuroaxonal damage, and reactive astrogliosis. The assessment was conducted in relation to clinical diagnosis and Aβ PET in a retrospective manner. The results of this study provide real-world evidence on the diagnostic utility of plasma biomarkers in clinical settings.

## Materials and methods

### Study design and participants

This study sample consisted of 126 patients (age = 65 ± 8.5 mean ± SD, 42–86 years (range), 70 female/56 male), who had undergone extensive cognitive assessment at the Clinic for Cognitive Disorders at Karolinska University Hospital in Stockholm, Sweden. The patients had been referred due to different cognitive complaints from primary care physicians (GPs) and, in a few cases, from other specialist or memory clinics for a second opinion.

### Diagnostic assessment

The clinical workup included physical, neurological, neuropsychological, and psychiatric assessments, medical history, CT/MR imaging, CSF biomarker analysis, apolipoprotein E (*APOE*) genotyping and, in some cases, [^18^F]FDG (fluorodeoxyglucose) PET. The majority of patients completed a multi-domain neuropsychological battery of tests [17].

Final diagnoses were achieved by consensus from a dementia expert team comprised of specialists in cognitive disorders, clinical neuropsychologists, and specialist nurses. The main diagnostic categories included MCI [18]; Alzheimer´s disease [19]; and non-Alzheimer´s disease dementias, including dementia of unclear aetiology (not otherwise specified; WHO, 1992); Lewy body dementia [20]; frontotemporal dementia [21]; vascular dementia including subcortical types [22]; primary age-related tauopathy [23, 24]; and alcohol-related dementia [25].

Subsequently for refining the diagnosis in patients with uninformative or contradictory biomarkers, the patients were referred to an [^18^F]flutemetamol PET (Aβ PET) examination that could take up to 1.5 years after the first extensive assessment for results. Nonetheless, this relatively short time lag should not constitute an issue for cross-sectional analyses since studies have reported that Aβ PET results remain relatively stable in even longer time periods: one of these studies was on older adults who were cognitively intact or who had MCI demonstrated and were scanned twice using [^18^F]flutemetamol over a period of 3.6 years [26]; in two clinical trial studies lasted around 75 weeks, the placebo arms did not result in significant changes in Aβ PET quantification [27, 28]. The results of the Aβ PET scans have been made available to the responsible physician and led to a change in diagnosis in a subset of individuals. [^18^F]Flutemetamol images were visually assessed as positive or negative by an experienced nuclear medicine physician (I.S.) and led, together with the clinical information available from the PET visit, to the CSF biomarker-based diagnoses: MCI Aβ– (n = 29), MCI Aβ + (*n* = 19), AD (*n* = 51), non-AD (*n* = 23), or cognitive unimpaired (CU) (*n* = 4). After this extensive clinical assessment, the diagnosis of neurodegenerative disorder was ruled out for four patients (these patients were then grouped and are referred to as CU individuals).

Medical records of MCI Aβ+ patients were revised by I.S and A.N. and for any change in their diagnosis. For 18 patients the information about the latest diagnosis (on 15.03.2022) has been retrieved and used in the present analysis as a subgroup with a follow-up.

Subjects’ consent was obtained according to the Declaration of Helsinki. The Regional Human Ethics Committee of Stockholm, Sweden, and the Isotope Committee of Karolinska University Hospital Huddinge approved this study. All patients gave their written informed consent.

### CSF collection and analysis

Samples of CSF were collected via standard lumbar puncture under non-fasting conditions. Sample analyses were performed at the Clinical Neurochemistry Laboratory, Sahlgrenska University Hospital, Mölndal, Sweden, where levels of Aβ1-42, tTau and pTau were determined using commercially-available sandwich ELISAs (Innogenetics, Ghent, Belgium).

### PET imaging

[^18^F]Flutemetamol PET scans were acquired using either a Biograph mCT PET/CT scanner (Siemens/CTI, Knoxville, TN) or GE Discovery scanner (General Electrics, USA) at the Department of Medical Radiation Physics and Nuclear Medicine Imaging, Karolinska University Hospital, Huddinge, Sweden, as detailed elsewhere [2]. Reconstruction of the [^18^F]flutemetamol PET images were obtained using point spread function (PSF) modelling and time-of-flight (ToF) algorithm (3 iterations, 21 subsets, 3.0 mm gaussian filter), resulting in the resolution of 128 × 128 x 1 (pixels), and a voxel size (mm) of 2.12 × 2.12 ×1. [^18^F]Flutemetamol summation images were visually assessed as positive or negative by an experienced nuclear medicine physician (I.S.) according to the product-specific guidelines. Additionally, PET images were pre-processed with the robust PET-only pipeline (rPOP) [29] for PET-only datasets in MATLAB (MathWorks, v.R2022_a) and SPM 12, and standardised uptake value ratios (SUVRs) were calculated using whole cerebellum as a reference region. Centiloids were calculated using an in-house centiloid calibration pipeline, based on the methods described in Klunk et al. [30] and Battle et al. [31].

### Plasma collection and analysis of plasma biomarkers

Plasma was collected into sodium-heparin tubes (Vacutainer®, BD Diagnostics) and centrifuged (1500 g, +4 °C) for 10 min. Following centrifugation, the samples were aliquoted into polypropylene tubes and stored at −80 °C within 30–60 min of collection. Aβ40, Aβ42, GFAP, and NfL were quantified in plasma using a multiplexed single molecule assay (SIMOA, N4PE from Quanterix). Plasma pTau181 and pTau231 were assessed using in-house developed SIMOA assays described in Karikari et al. [32], and Ashton et al. [33], respectively.

### Statistical analysis

Statistical analysis was carried out in R (version 1.4.1717, https://www.r-project.org), while data visualisations were created using ggplot2 (v3.3.5). Sex differences were tested using Pearson’s chi-squared test, and differences in continuous variables were tested using the Kruskal–Wallis one-way analysis of variance (‘stats’, v4.1.1) and Dunn’s post hoc test for pairwise comparisons after applying the false discovery rate (FDR) correction for multiple comparisons (‘rstatix’, v0.7.0). The CU group was excluded from group comparisons due to its small size (*n* = 4). Because of the non-normal distribution of the variable levels, relationships between plasma biomarkers and the Aβ PET burden were tested using the Spearman’s rank correlation coefficient (‘correlation’, v0.8.0). No patients were excluded from the sample and the number of patients per groups can be found in the Table 1.

**Table 1:** General characteristics and plasma biomarkers across diagnostic groups.

	MCI Aβ– (*n* = 29)	MCI Aβ + (*n* = 19)	AD *(n* = 51)	Non-AD (*n* = 23)	CU (*n* = 4)	Total (*N* = 126)	*p* value
Age							0.831 (1)
Mean (SD)	66.03 (10.81)	66.53 (8.28)	64.12 (7.26)	65.70 (8.74)	64.00 (2.16)	65.21 (8.47)	
Min – Max	44.00–86.00	54.00–80.00	48.00–83.00	42.00–81.00	62.00–67.00	42.00–86.00	
Sex							0.148 (2)
F	16 (55.2%)	15 (78.9%)	28 (54.9%)	9 (39.1%)	2 (50.0%)	70 (55.6%)	
M	13 (44.8%)	4 (21.1%)	23 (45.1%)	14 (60.9%)	2 (50.0%)	56 (44.4%)	
MMSE*							<0.001 (1)
Mean (SD)	25.55 (3.42)	27.42 (1.89)	25.38 (3.38)	23.32 (4.04)	29.50 (0.58)	25.50 (3.53)	
Min – Max	17.00–30.00	24.00–30.00	17.00–30.00	15.00–29.00	29.00–30.00	15.00–30.00	
APOE ε4 (%)	7 (35.0%)	9 (64.3%)	30 (85.7%)	3 (18.8%)	1 (25.0%)	50 (56.2%)	<0.001 (2)
Missing	9	5	16	7	0	37	
Centiloid**							<0.001 (1)
Mean (SD)	−2.22 (14.41)	77.75 (25.32)	96.11 (26.91)	−0.72 (18.71)	−7.19 (2.42)	50.23 (51.77)	
Min – Max	−23.47–37.72	33.37–121.39	37.74–162.25	−37.67–42.87	−9.97–4.84	−37.67–162.25	
CSF Aβ42 (pg/ml)							<0.001 (1)
Mean (SD)	673.28 (177.82)	597.00 (127.64)	522.69 (149.56)	738.43 (335.57)	1090.75 (354.90)	625.97 (235.49)	
Min – Max	389.00–1140.00	256.00–830.00	281.00–870.00	280.00–1650.00	796.00–1540.00	256.00–1650.00	
CSF tTau (pg/ml)***							<0.001 (1)
Mean (SD)	246.48 (189.58)	517.89 (197.38)	522.16 (229.75)	316.26 (241.04)	193.25 (33.36)	409.14 (247.42)	
Min – Max	75.00–1060.00	280.00–1130.00	180.00–1290.00	86.00–1120.00	150.00–229.00	75.00–1290.00	
CSF pTau (pg/ml)							<0.001 (1)
Mean (SD)	34.38 (13.22)	71.74 (29.81)	76.12 (39.63)	43.00 (22.76)	31.00 (11.34)	58.37 (35.30)	
Min – Max	15.00–77.00	15.00–150.00	24.00–240.00	16.00–118.00	16.00–43.00	15.00–240.00	
Aβ40 (pg/ml)							0.112 (1)
Mean (SD)	114.09 (34.60)	112.44 (16.07)	111.72 (33.37)	126.05 (25.80)	110.64 (35.27)	114.95 (30.44)	
Min – Max	7.84–177.96	79.35–132.09	2.26–247.50	95.82–213.36	82.63–162.22	2.26–247.50	
Aβ42 (pg/ml)							0.010 (1)
Mean (SD)	6.63 (2.30)	6.45 (1.46)	6.12 (1.99)	7.85 (1.90)	7.41 (1.84)	6.65 (2.05)	
Min – Max	0.46–11.06	3.45–8.61	0.57–13.43	5.52–13.69	5.22–9.72	0.46–13.69	
Aβ42/40							0.059 (1)
Mean (SD)	0.06 (0.01)	0.06 (0.01)	0.06 (0.03)	0.06 (0.01)	0.07 (0.02)	0.06 (0.02)	
Min – Max	0.03–0.08	0.03–0.07	0.03–0.25	0.04–0.08	0.05–0.09	0.03–0.25	
GFAP (pg/ml)							<0.001 (1)
Mean (SD)	97.76 (47.86)	151.70 (53.16)	175.22 (74.09)	125.70 (62.68)	69.85 (17.41)	141.46 (70.35)	
Min – Max	35.36–264.36	62.10–280.38	60.24–392.18	43.47–296.02	53.79–90.16	35.36–392.18	
NFL (pg/ml)							0.033 (1)
Mean (SD)	22.85 (16.56)	18.99 (6.37)	22.80 (10.09)	24.61 (12.15)	10.75 (3.14)	22.18 (11.89)	
Min – Max	6.74–89.89	9.00–31.22	7.49–49.80	12.34–60.21	8.24–15.21	6.74–89.89	
pTau181 (pg/ml)							<0.001 (1)
Mean (SD)	10.78 (5.87)	10.50 (4.04)	16.91 (20.18)	13.69 (18.19)	7.17 (0.71)	13.64 (15.50)	
Min – Max	3.31–30.00	4.68–23.53	4.44–151.38	3.95–95.30	6.47–7.90	3.31–151.38	
pTau231 (pg/ml)							<0.001 (1)
Mean (SD)	13.60 (5.77)	15.58 (6.26)	18.84 (7.77)	14.87 (6.55)	8.65 (1.89)	16.10 (7.17)	
Min – Max	1.93–27.72	8.28–36.72	3.16–42.18	5.48–27.72	6.90–11.34	1.93–42.18	
pTau181/Aβ42							<0.001 (1)
Mean (SD)	1.94 (1.54)	1.76 (1.08)	3.38 (6.33)	1.83 (2.56)	1.00 (0.20)	2.45 (4.30)	
Min – Max	0.50–7.23	0.67–5.81	0.74–46.36	0.55–13.32	0.81–1.27	0.50–46.36	

*MMSE scores were missing for 1 AD and 1 Non-AD.

**Centiloid value was missing for 1 AD.

***CSF tTau value was missing for 1 AD.

(1). Kruskal**–**Wallis rank sum test

(2). Pearson’s chi-squared test

Visual assessment of Aβ PET status by the nuclear medicine physician (I.S.) was used as a dichotomised predicted outcome in the ROC analyses. To identify the combination of biomarkers best able to predict Aβ PET status as defined via Aβ PET, we implemented the regression model with least absolute shrinkage and selection operator (LASSO) (‘glmnet’, v4.1.4), which was validated using tenfold cross-validation to determine the optimal LASSO penalty. We chose this analysis as it allowed us to maximise the discrimination accuracy of the model as well as reduce its dimensions by avoiding overfitting and dropping redundant variables. A total of 10 variables were included in the LASSO regression: plasma GFAP, NfL, pTau181, pTau231, Aβ42/Aβ40 ratio, Aβ42, Aβ40, pTau181/Aβ42, age, and sex. The performances of the best LASSO model and single biomarkers were compared by ROC curve analysis (‘pROC’, v1.18.0). The Youden index was used to obtain the optimal biomarkers’ thresholds (as per pROC defaults). The performance of the plasma biomarkers in predicting Aβ PET status was tested in the whole cohort and in a subgroup of 75 MCI patients classified as such before knowledge of the Aβ PET result. The performance of the plasma biomarkers in predicting conversion from MCI Aβ+ to AD for 18 patients, for whom follow-up diagnoses were available, was also tested. A missing value, for any variable, resulted in the exclusion of the patient for the specific sub-analysis that required that variable. A *p*-value of 0.05 was regarded as significant.

## Results

### Sample cohort and demographics

Demographic and biomarker data for all participants are shown in Table 1. In total, the study population consisted of 126 individuals with plasma biomarker measurements and Aβ PET scans. The study’s diagnostic groups were not significantly different in terms of age (*p* = 0.81) or sex (*p* = 0.18) distribution. The MMSE scores were significantly different between the groups (*p* < 0.001) and the following pair-wise comparisons produced significant results in the posthoc analysis upon FDR correction: MCI Aβ– > non-Alzheimer´s dementias; MCI Aβ– < CU; MCI Aβ + > Alzheimer´s disease dementia and non-Alzheimer´s dementias; Alzheimer´s disease dementia > non-Alzheimer´s dementias.

### Plasma biomarkers across diagnostic groups

**Fig. 1:**
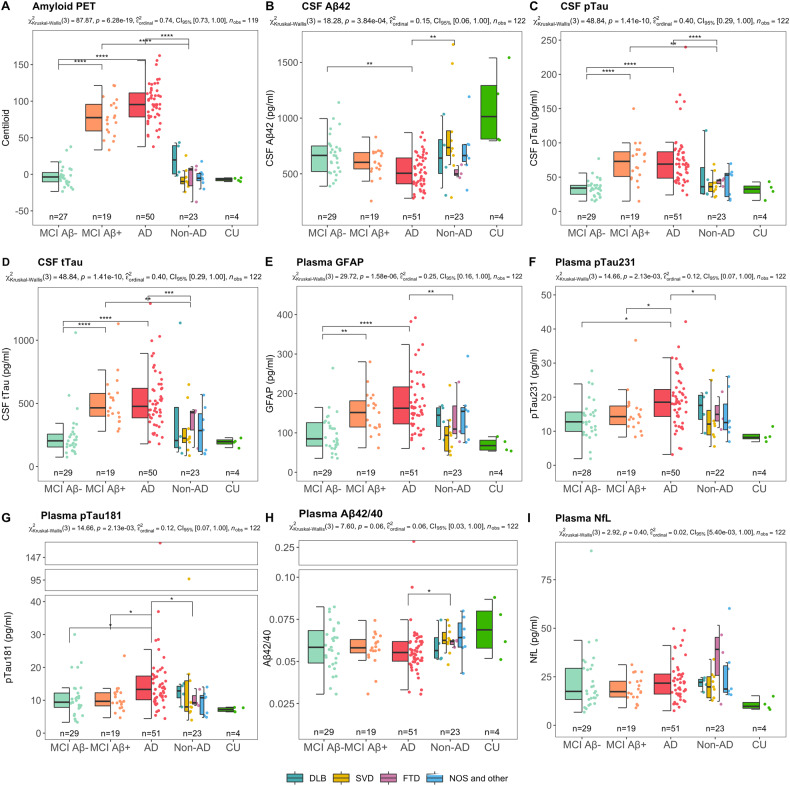
Levels of Aβ PET (in centiloid), and CSF Alzheimer’s disease biomarkers by diagnostic group. **A** PET centiloid was significantly higher in patients diagnosed on AD spectrum. **B** CSF Ab42 was decreased in patients with AD dementia in comparison to the MCI Aβ– and non-AD dementia groups, whereas no significant difference could be found between MCI Aβ– and Aβ+ groups. In contrast, in the given cohort, levels of CSF pTau and tTau (plots **C** and **D**, respectively) were significantly elevated in patients diagnosed on the AD spectrum in relation to patients with non-AD-related pathologies (including comparison between MCI Aβ– and Aβ+ individuals). **E** Plasma GFAP were statistically different between the groups with minimal (non-AD), intermediate (MCI Aβ + ), and high levels (AD) of Aβ pathology. **F**, **G** pTau231 and pTau181 were statistically different between MCI Aβ– and AD in relation to non-AD. **H**, **I** No statistically significant difference between the diagnostic groups was observed for plasma NfL and Aβ42/40, except for a significant decrease in plasma Aβ42/40 in AD compared non-AD dementia group. The p values in the subtitles indicate the results of analysis of variance with the Kruskal–Wallis test, between groups, and of post-hoc analysis with Dunn’s test and multiple comparisons correction with the false discovery rate. *p < 0.05, **p < 0.01, ***p < 0.001, ****p < 0.0001.

Figure 1 shows the box plots of the biomarkers across the five diagnostic groups. PET centiloid values, CSF-pTau, and CSF tTau (Fig. 1A, C, D) were significantly higher in patients on the AD spectrum (MCI Aβ+ or AD) compared to MCI Aβ– or non-AD groups (*p* < 0.0001, for all); while, in our cohort, CSF Aβ42 (Fig. 1B) was only significantly reduced in the AD group compared with MCI Aβ– and non-AD groups (p < 0.01, for both), but there was no difference between MCI Aβ+ and MCI Aβ–. It is of note that since some patients included in this study have been referred to Aβ PET because of conflicting CSF Aβ and pTau/tTau biomarker profiles, the study sample is enriched with patients with discordant CSF/PET Aβ results. Among the plasma biomarkers, only GFAP levels were significantly higher in the MCI Aβ+ compared to the MCI Aβ– group (Fig. 1E), while plasma pTau181, pTau231, and pTau181/Aβ42 ratio levels were different between MCI Aβ+ and AD (higher in AD) (Fig. 1E, G, H). Plasma GFAP, pTau181, pTau231 were also elevated in AD compared to MCI Aβ– and non-AD groups, while the concentration of plasma NfL and plasma Aβ42/40 ratio were not statistically different across the diagnostic groups (Fig. 1H, I).

### Plasma biomarkers in association with amyloid PET (centiloid)

**Fig. 2:**
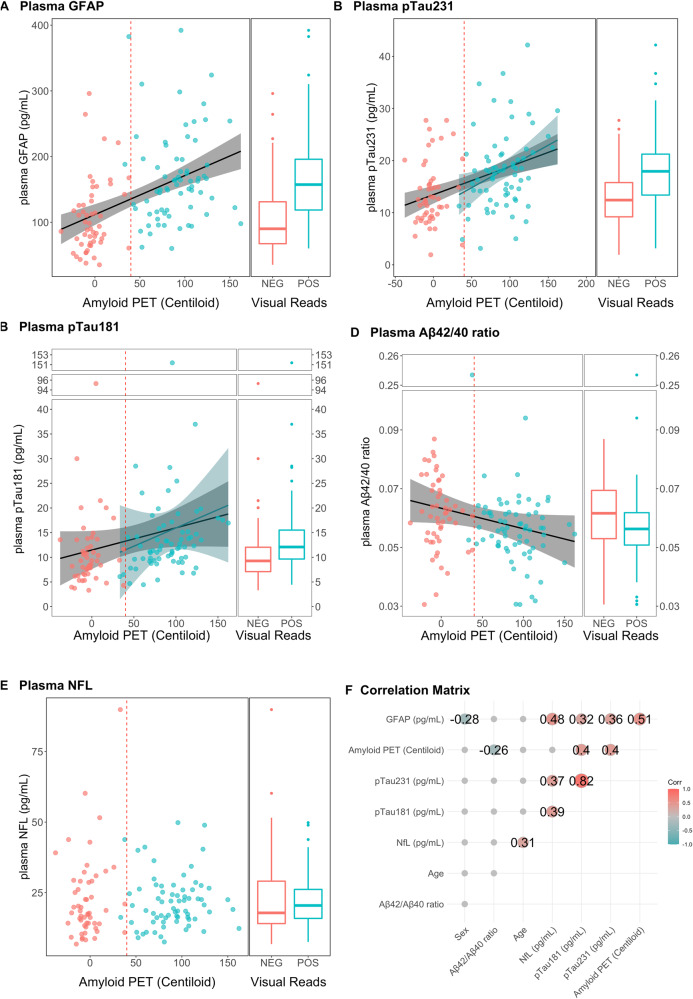
Linear Regressions between plasma biomarkers and Aβ PET (in centiloid) in the whole dataset. **A** Plasma GFAP was positively associated with Aβ accumulation in the brain in the whole dataset and in the amyloid-positive and amyloid-negative groups, separately. **B**, **C** Plasma pTau181 (**B**) and pTau181 (**C**) were positively associated with Aβ accumulation in the brain in the whole dataset and in the amyloid-positive groups. **D** Plasma NfL was not related to Aβ PET in any group. **E** Plasma Aβ42/40 was negatively associated with Aβ PET in the whole group only. **F** Regression lines are drawn only if the Spearman’s rho statistic was significant (*p* < 0.05). Correlation matrix showing the Spearman’s rho coefficients for the associations between the variables considered.

**Fig. 3:**
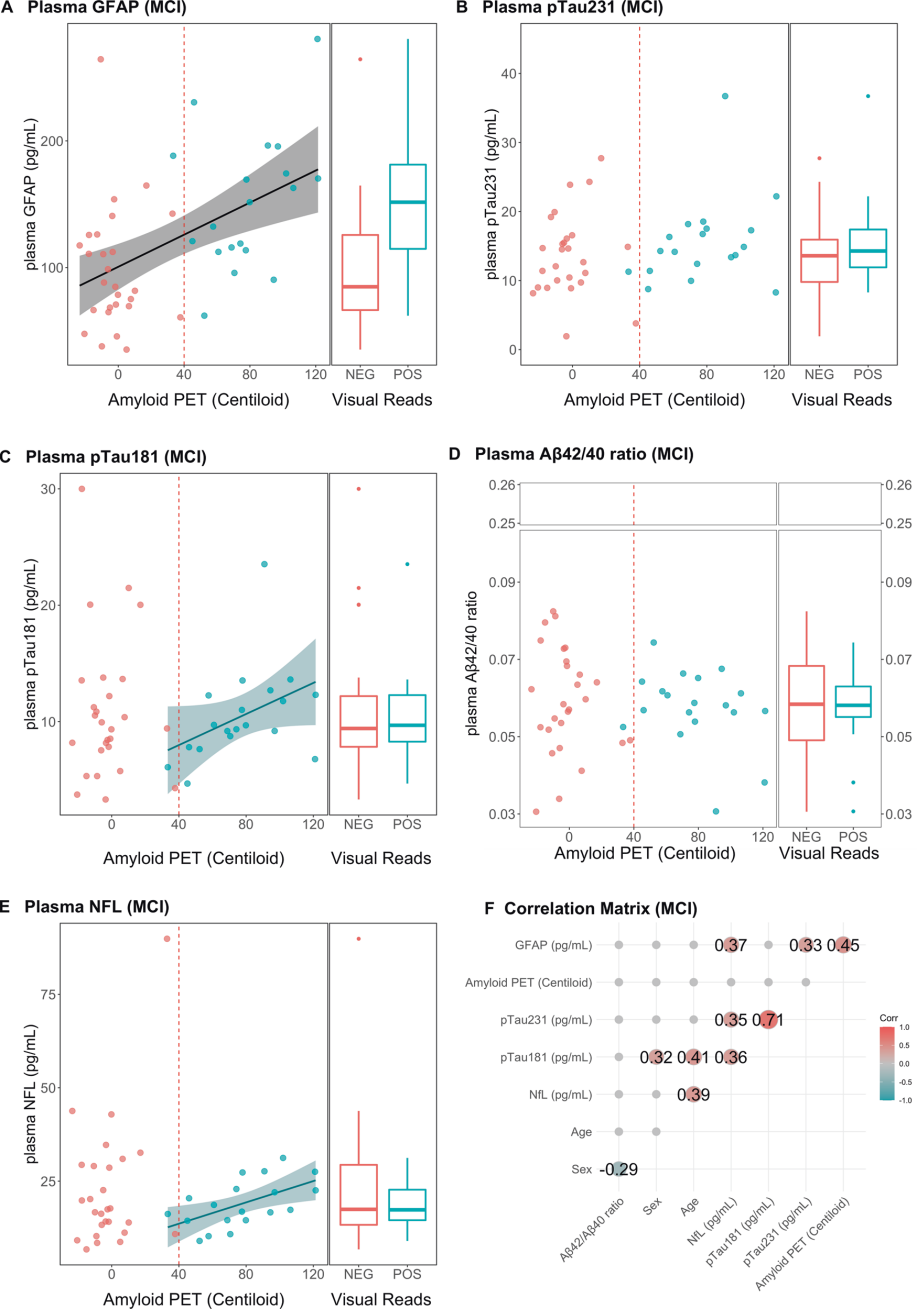
Linear Regressions between plasma biomarkers and Aβ PET (in centiloid) in MCI group. **A** Plasma GFAP was the only biomarker positively associated with amyloid accumulation in the brain in the MCI group. **B** Plasma pTau181, (**C**) pTau231, **D** plasma NFL and (**E**) plasma Aβ42/40 were not related with amyloid PET in the MCI group. **C** pTau231 was the only biomarker positively associated with amyloid accumulation in the brain in the MCI Aβ+ group. **F** Regression lines are drawn only if the Spearman’s rho statistic was significant (*p* < 0.05). Correlation matrix showing the Spearman’s rho coefficients for the associations between the variables considered.

To further corroborate the group comparison results, correlation analyses of plasma biomarkers were performed with Aβ PET centiloid values in a continuous fashion. The correlations between Aβ PET (centiloid, CL) and plasma biomarkers and the group comparison between PET visual positive and negative (in the subpanels on the right) are depicted in Fig. 2 (whole dataset) and Fig. 3 (MCI only).

Significantly higher plasma values of GFAP, pTau181, pTau231, and lower levels of Aβ42/40 ratios were observed in Aβ+ compared to Aβ– patients, whereas no significant difference in plasma NfL values was observed between Aβ+ and Aβ– (Fig. 2), but in the MCI subset only plasma GFAP was elevated in the Aβ+ group compared to Aβ– group (*p* < 0.0001) (Fig. 3A). Accordingly, the described group differences were accompanied with significant linear relationships between Aβ PET (centiloid) and plasma biomarkers (Figs. 2 and 3). Notably, plasma GFAP in the whole dataset had significant association (rho =0.52, *p* < 0.001), while a trend could be observed in the Aβ+ group (rho =0.21, *p* = 0.07), and in the Aβ– group (rho =0.26, *p* = 0.06) (Fig. 2A). Plasma pTau181 and pTau231 had significant associations with Aβ PET (centiloid) in the whole dataset (rho =0.40, *p* < 0.0001, for both) but also in the Aβ+ subgroup (rho =0.34*, p* < 0.001 and rho =0.24, *p* = 0.02, respectively) (Fig. 2B, C). Plasma Aβ42/40 ratio levels were negatively associated with Aβ PET (centiloid) (rho =−0.26, *p* < 0.01). In the MCI group, besides the association between GFAP and Aβ PET (centiloid) (rho =0.45, *p* < 0.001) (Fig. 3A) that reflects the group difference (in the same direction), Aβ PET had a significant relationship between plasma pTau231, pTau181 and plasma NfL in the Aβ+ group only (rho =0.29, rho =0.56 and rho =0.61, respectively, *p* ≤ 0.02, for all) (Fig. 3B, C, E).

### ROC analyses for predicting amyloid positivity and conversion to AD

**Fig. 4:**
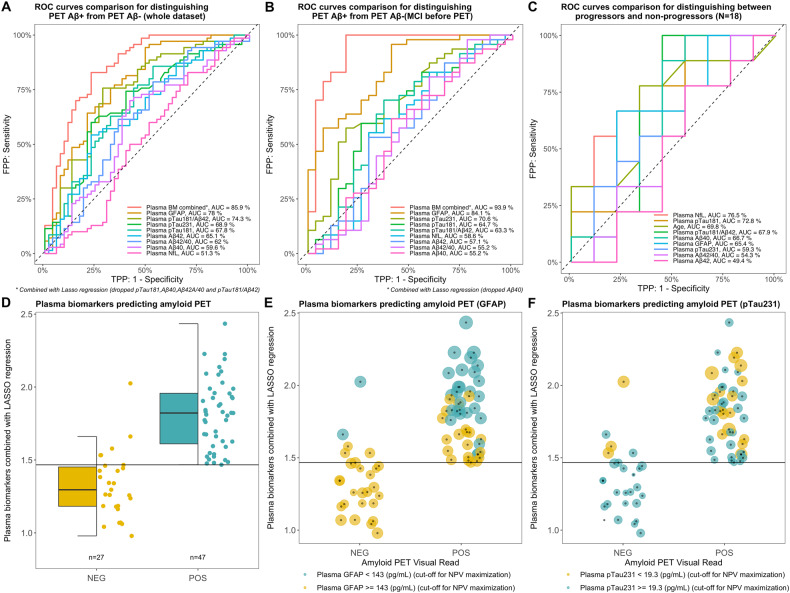
Plasma biomarkers as predictors for amyloid PET visual read positivity and conversion to AD. The combination of biomarkers obtained by LASSO regression first, and plasma GFAP as a single biomarker second, resulted in the two largest AUCs for predicting Aβ positivity in the whole dataset (**A**) and in the prior to PET MCI group (**B**). **C** Plasma NfL was the best predictor for conversion to AD in the MCI Aβ+ group. **D**, **E**, **F** Visualisation of the pooled variable obtained via LASSO regression for the prior to PET MCI group that resulted the best AUC (and specificity and negative predictive values of 100%) from panel (**B**). **D** No false positives were identified by the pooled variable. **E**, **F** Plasma GFAP and pTau231 values are visualised according to point size, and the colour of the balloons is determined by a threshold for maximisation of the negative predictive value (NPV) (having a test with no false negatives); 143 (pg/ml) and 19.3 (pg/ml) for GFAP and pTau231, respectively.

To evaluate the discriminatory capacity of the plasma biomarkers to detect amyloid positivity in the clinical setting, we performed ROC curve analyses. When plasma biomarkers were tested separately, in both the whole dataset (Fig. 4A, Table 2A) and the MCI group (prior to PET) (Fig. 4B, Table 2B), plasma GFAP showed the best performance in discrimination between Aβ PET status as defined by Aβ PET with AUCs of 78% and 84% respectively. Also, plasma GFAP was characterised by a high sensitivity (94% and 96%, respectively, Table 2A–B), but a low specificity (51% and 59%, respectively). When plasma biomarkers were tested in combination via a LASSO regression, we found that in the whole dataset, the model with the best result dropped pTau181, Aβ40, Aβ42/Aβ40, and pTau181/Aβ42, nonetheless resulting in the higher AUC (86%) than for any plasma biomarker analysed separately. In the group of patients diagnosed with MCI, plasma biomarkers combined via LASSO regression resulted in an AUC of 97% with a sensitivity of 100%, a specificity of 81.5%, a positive predictive value of 92%, and a negative predictive value of 100%. Figure 4D illustrates that in the MCI patients (prior to PET) the optimal cut-off point for the panel of biomarkers combined via LASSO regression produced no false negatives and 5 false positives (out of 27 total positive) when discriminating between amyloid-negative and amyloid-positive individuals. The false positives have been further investigated (more details are available in Supplementary Table 1), and as shown in Fig. 4E (GFAP) and 4F (pTau231) these patients have high values in GFAP and pTau231 (each circle’s size is proportional to the value of the plasma biomarker after scaling and centring), which were also the major drivers of the pooled combinatory variable (Supplementary Table 2). In Fig. 4E and F the circles are two colours based on an a posteriori cut-off value calculated to maximise the negative predictive value (hence reducing the false negative to zero) in relation to the pooled variable, and the values obtained were 143 pg/ml and 19.3 pg/ml for GFAP and pTau231, respectively.

**Table 2:** ROC analyses to discriminate Aβ PET-positive from Aβ PET-negative individuals.

Cohort	Predictor	AUC (%)	95% CI	Cut-off	Specificity (%)	Sensitivity (%)	PPV*	NPV**
A: Discriminatory performance of plasma biomarkers: ROC curves analysis								
Entire cohort (n =123†)	BMs combined via Lasso regression (dropped pTau181 and Aβ40, and pTau181/Aβ42 ratio)	85.9	79–92.8	1.5	77.4	82.9	83	77
	Plasma GFAP	78.0	69.5–86.4	90.3	50.9	94.3	72	87
	Plasma pTau231	68.9	59.3–78.5	16.1	77.4	60.0	78	59
	Plasma pTau181/Aβ42	74.3	65.2–83.4	1.6	71.7	75.7	78	69
	Plasma pTau181	67.8	58.1–77.6	8.6	47.2	85.7	68	71
	Plasma Aβ42	65.1	55.2–75.1	6.4	77.4	54.3	76	56
	Plasma Aβ42/40	62.0	51.6–72.4	0.1	47.2	78.6	66	62
	Plasma Aβ40	59.6	49.1–70	117.9	58.5	70.0	69	60
	Plasma NFL	51.3	40.6–62.1	14.3	30.2	82.9	61	57
B: Discriminatory performance of plasma biomarkers: ROC curves analysis								
Subset of individuals with MCI (n =74††)	BMs combined via Lasso regression (dropped Aβ40)	93.9	87.2–100	1.5	81.5	100.0	90	100
	Plasma GFAP	84.1	75–93.2	90.3	59.3	95.7	80	89
	Plasma pTau231	70.6	58.1–83.1	15.4	81.5	57.4	84	52
	Plasma pTau181/Aβ42	63.3	49–77.5	1.6	63.0	70.2	77	55
	Plasma pTau181	64.7	50.8–78.6	10.9	74.1	59.6	80	51
	Plasma Aβ42	57.1	42.6–71.5	6.5	70.4	53.2	76	46
	Plasma Aβ42/40	55.2	40.2–70.3	0.1	40.7	80.9	70	55
	Plasma Aβ40	55.2	40.6–69.7	116.8	59.3	61.7	72	47
	Plasma NFL	58.6	43.7–73.4	20.3	70.4	55.3	76	48
C: Discriminatory performance of plasma biomarkers: ROC curves analysis								
Subset of individuals with follow-up (n = 18)	Lasso regression (kept only NfL-> see NfL)	–	–	–	–	–	–	–
	Plasma GFAP	65.4	37.3–93.5	142.0	77.8	66.7	75	70
	Plasma pTau231	59.3	31.1–87.4	17.0	44.4	77.8	58	67
	Plasma pTau181/Aβ42	67.9	39.3–96.5	1.9	55.6	100.0	69	100
	Plasma pTau181	72.8	47.9–97.8	12.5	44.4	100.0	64	100
	Plasma Aβ42	49.4	20.1–78.7	7.2	44.4	77.8	58	67
	Plasma Aβ42/40	54.3	25.5–83.1	0.0	22.2	100.0	56	100
	Plasma Aβ40	66.7	38.8–94.5	128.4	44.4	100.0	64	100
	Plasma NFL	76.5	53.6–99.5	25.1	44.4	100.0	64	100

*PPV = positive predictive value. **NPV = negative predictive value. ^†^pTau231 missing in 3 subjects. ^††^pTau231 missing in 3 subjects.

Sensitivity = probability of detecting true positive (100% if all true positives (TP) are detected and no false negatives (FN) are present).

Specificity = probability of detecting true negative (100% if all true negatives (TN) are present and no false positive (FP) are present).

Positive Predictive Value (PPV) = probability of true positive test (100% if only true positives (TP) are detected and no false positives (FP) are present).

Negative Predictive Value (NPV) = probability of true negative test (100% if only true negatives (TN) are detected and no false negatives (FN) are present).

For a limited number of patients, follow-up diagnoses were available, allowing us to perform a ROC analysis on the conversion to AD (n = 9/18) for the MCI Aβ + (Fig. 4C). In this case, plasma NFL resulted the highest AUC (0.77) with the smallest 95% CI range and a sensitivity of 100%, but a low specificity of 44.4%. The LASSO regression identified NfL among all variables entered as the best predictive variable (results not shown).

## Discussion

In the present study, we found that in the MCI patients from the tertiary memory clinic, the panel of plasma biomarkers had superior performance discriminating between amyloid-positive and amyloid-negative patients, as defined by Aβ PET (visual reads), in comparison to single plasma biomarker performance. According to the ROC analysis in the MCI group (prior to any knowledge derived from PET), the sensitivity and negative predictive value of the plasma biomarker panel were 100%, resulting in no false negatives. In other words, all patients with amyloid-negative PET scans were detected as such by the combined application of plasma biomarkers (GFAP, NfL, pTau231, pTau181, and pTau181/Aβ42, and Aβ42/Aβ40 ratios). This observation must be considered important in view of a possible screening perspective.

In the present study we compared plasma biomarkers across the clinical diagnostic groups and found that plasma GFAP levels were higher in the MCI Aβ+ group compared to the MCI Aβ– group. This is in line with what has been found previously in a clinical cohort of stable and unstable MCI patients stratified according to CSF Aβ42 [34], and some MCI research cohorts stratified according to Aβ PET. To this day, it is still unclear to which extent, but it is known that peripheral cells contribute to plasma GFAP concentrations [35]; it is also unclear which brain pathological process is reflected in plasma GFAP concentrations. Plasma GFAP relationship with GFAP-positive reactive astrocytes has not been proved and while many claim that it could be a marker of amyloid deposition, plasma GFAP is detectable even in other dementia disorders [36]. Also, plasma pTau181 and pTau231 had higher values in the AD group compared to the MCI Aβ+ group. In research cohorts, plasma pTau181 and pTau231 were found to be the best predictors of amyloidosis in cognitively-impaired patients [8, 15, 33]. In line with these findings, we extend this notion to a clinical cohort at a tertiary memory clinic. Therefore, at first glance, our findings suggest that plasma GFAP is an earlier biomarker of AD compared to plasma pTau181 and pTau231, while plasma pTau181 and pTau231 seem to better capture pathological changes associated with amyloidosis at later stages of the disease possibly since sometimes plasma GFAP levels decline in advances AD stages [37].

Our results show that plasma GFAP was the only biomarker in the MCI group with a positive association with Aβ PET centiloids. In the whole cohort, most of the investigated plasma biomarkers (i.e., GFAP, pTau181, pTau231, and Aβ42/Aβ40 ratios) were associated with [^18^F]flutemetamol uptake but not plasma NfL. In a previously published research cohort study, the relationship between Aβ PET and plasma GFAP in cognitively unimpaired and impaired groups has already been documented [38]. We extend this evidence to a clinical cohort, and we also focus on the AD spectrum, finding a positive association within the MCI subgroup. GFAP is known as a marker of reactive astrogliosis based on post-mortem brain immunostaining studies, but how plasma GFAP levels correlate to reactive brain astrogliosis is still under investigation. Recent studies however suggest that brain amyloidosis is linked to the levels of GFAP protein in plasma [39]. This suggestion is in accordance with the recent results obtained from a clinical trial (TRAILBLAZER-ALZ) showing that plasma GFAP (and pTau217) decreased after anti-amyloid treatment that decreased plaque accumulation in the brain [40].

To evaluate the utility of plasma biomarkers in clinical settings, we compared the performance of single and combined applications of these biomarkers in discriminating between amyloid-positive and amyloid-negative individuals, as defined by visual read of Aβ PET scans. In the whole cohort and in the MCI group, plasma GFAP (followed by plasma pTau231) was the best single predictor for amyloid positivity. This is also supported by findings in research cohorts where plasma GFAP was a predictor of amyloidosis, especially in preclinical cohorts [8, 15].

It must be noted that plasma GFAP, in the whole dataset as well as in the MCI group, had low specificity (51% and 59%, respectively), and accordingly moderately positive (72% and 80%, respectively) and negative predictive values (87% and 89%, respectively). It follows, that by relying solely on the GFAP values in a hypothetical cohort, 20/100 MCI patients would be false positives and 11/100 would be false negatives. Similar results have been found in the TRIAD research cohort, which spans the AD spectrum. In this study, GFAP had an AUC of 0.85 (compared to our 0.78) in predicting Aβ PET positivity (visual reads), but specificity and sensitivity were not reported for this analysis [41]. It is expected that performance declines in the clinical setting with heterogeneous cohorts. On the other hand, combining information from several biomarkers resulted in a negative predictive value of 100% in the MCI group. These results suggest that the best combination of biomarkers may be useful in a rule-out diagnostic algorithm. A negative test result (from the pooled variable) translates in a minimal (absent in our cohort) risk of having a positive Aβ PET scan from a visual reading. There were only five false positives according to the pooled variable, and, as shown in Fig. 4E, F, the false positives were characterised by high values of GFAP and pTau231 that were, in fact, the major contributors to the pooled variables. Importantly, the best results were obtained in the MCI group after thorough clinical assessment (but before PET assessment) and the results from the whole cohort resulted in a sensitivity of 83% and specificity of 77%.

Although earlier reports suggested plasma Aβ42/40 ratios as a reliable marker of Aβ pathology [8, 11], in our study levels of Aβ42/40 were not different between MCI Aβ–, MCI Aβ+ and AD groups, and showed only week correlation with [^18^F]flutemetamol uptake. Three factors might have contributed to this discrepancy. First, studies reporting strong and reliable performance of Aβ42/40 ratios in plasma used mass spectroscopy, whereas in the present study SIMOA assays were used to measure levels of Aβ42 and Aβ40 proteins. When mass spectroscopy and immunoassays results were compared in the same samples, immunoassays showed 20% lower areas under the ROC curve [42]. In line with this, studies employing this method to measure Aβ42/40 ratios often report AUC of 60%–70% for identifying Aβ status [14, 16, 43–45]—similar to what we have observed in the present study. Secondly, researchers have noted that since the overall Aβ42/40 change is small (compare to these observed in CSF) preanalytical and analytical variability or errors could significantly affect the robustness of the test [8, 46].

For a small subset of the cohort, data from the follow-up diagnoses were available, and for these patients (18 MCI Aβ +) it was possible to evaluate the predictive power of the plasma biomarkers in detecting conversion to AD. In this group, 50% of patients converted to AD, and plasma NfL resulted as the plasma biomarker with the highest AUC and smallest 95% CI range in predicting conversion to AD. This can be since NfL is a marker of neurodegeneration and is therefore indicative of a more advanced stage of the disease [47]. Our findings are in line with the earlier reports of both plasma NfL and plasma pTau181 as important predictors for conversion to AD from MCI over a four-year period [48].

One of the strongest aspects of the present study is that it is one of only a few studies in a real-world clinical setting. Research cohorts have strict inclusion and exclusion criteria, which translates into a higher degree of homogeneity than that in the general population and leaves out certain patients that end up being underrepresented in research cohorts.

According to a recently-published roadmap, plasma biomarkers can be employed for clinical trial pre-screening and only used as prescreening tool in the clinic, as no diagnoses should be based solely on their results; but combined with either PET or CSF results [49]. It is true that a blood sample to measure plasma biomarkers is easily obtained and could potentially cut costs and time from clinical practice, but we need to evaluate plasma biomarkers in all their aspects (for example, with longitudinal studies on cognition decline) and the laboratories that perform these measurements are still few in number and the biomarkers are still in validation phase at the individual level, limiting their use in clinical routines. To support this, a recent report from the EU/US CTAD task force highlights a lack of robustness in cut-offs left unvalidated for real-world cohorts [50]. Our results hint towards the possibility of using a combination of biomarkers to specifically rule out patients at low risk of having high levels of amyloidosis in the brain with a consequent risk of disease progression, but as is mentioned in the previous report, this should be performed primarily on cognitively-impaired patients.

### Limitations

The patients included in the present study were referred to a clinical Aβ PET investigation because their diagnosis was considered uncertain, including cases where CSF biomarker profile did not provide confidence (i.e., it was discordant) in diagnosing a patient as having either amyloid-negative or amyloid-positive MCI (prodromal AD), and in some cases the available clinical information made it difficult to discriminate between AD dementia and other non-AD forms of dementia such as FTD, LBD and vascular dementia. Nonetheless, with our study we have been able to investigate plasma biomarkers in a more realistic uncontrolled clinical setting, with a heterogeneous cohort of patients pooled from clinical practice.

Due to the small sample size of our longitudinal dataset, the conclusion drawn from our ROC analysis for discrimination between progressors versus non-progressors should be treated with caution.

Also, several studies noted the importance of correcting for *APOE* status, since adding this information improves the performance of the Aβ42/40 ratio in identifying Aβ status [10, 42, 43, 51–53]. Due to the lack of this information for 30% of the study population, this was not implemented in the current study since APOE status is not routinely clinically performed but only in selected cases.

Although our plasma marker panel was extensive, pTau217 was not measured. The latter has been found to be highly predictive for progression to AD in combination with other factors [54]. It will be of interest in the future to also evaluate plasma pTau217 performance in a real-world setting.

## Conclusions

In a retrospective clinical cohort, plasma biomarkers (GFAP, pTau181, pTau231, Aβ42/40 ratio) were significantly different between patients with dementias of AD and non-AD types. In line with the previous reports, plasma GFAP appears to be promising in discriminating between amyloid-positive and amyloid-negative MCI individuals. Most of the investigated biomarkers were associated and moderately predictive of Aβ deposition in the brain. Plasma GFAP seemed to increase early in disease progression, and be associated with amyloidosis, particularly in the MCI group. Despite this association, when examined separately, biomarkers showed weak-to-moderate performance in discriminating between negative or positive visual reads of Aβ PET scans (expect for GFAP, which showed good discriminatory power in the cohort of patients diagnosed with MCI). Nonetheless, especially in the MCI group, the combination of the different plasma biomarkers showed excellent ability to detect Aβ negative visual reads suggesting their potential role as rule-out tools in clinical practice. Prospective and longitudinal studies in real-world settings are warranted to replicate these results, develop algorithms, and refine diagnostic panels with the best discriminatory performance.

## Supplementary information

Supplementary Table 1–2

## Data Availability

The data that support the findings of this study are not publicly available, in order to maintain the privacy of research participants. The data are, however, available from the corresponding author upon reasonable request.

## Code availability

Standard R libraries were used to analyse the data. Code can be shared upon request.
