# Supplementary material for: Profiling of plasma biomarkers in the context of memory assessment in a tertiary memory clinic
Source: Transl Psychiatry. 2023 Jul 25;13:268. doi: 10.1038/s41398-023-02558-4 (PMC10368630; doi:10.1038/s41398-023-02558-4)
Supplement: Supplementary file 1 — Supplementary Table 1–2 [file 41398_2023_2558_MOESM1_ESM.docx]

| **False positive** | **Age** | **ApoE4** | **Diagnosis pre-PET** | **Diagnosis post-PET** | **MMSE** | **pGFAP** | **pTau231** | **pTau181** | **pNfL** | **pAβ42/40** | **Converted to AD** | **notes** |
| --- | --- | --- | --- | --- | --- | --- | --- | --- | --- | --- | --- | --- |
| 1 | >65 yrs | Carrier | MCI | MCI Aβ– | ≧27 | Q3 | Q4 | Q4 | Q2 | Q2 | No | - |
| 2 | ≦65 yrs | Carrier | MCI | MCI Aβ– | <27 | Q4 | Q3 | Q2 | Q3 | Q1 | No | severe depression with psychotic symptoms |
| 3 | ≦65 yrs | Carrier | MCI | MCI Aβ– | ≧27 | Q2 | Q4 | Q4 | Q4 | Q4 | No | (later in life) cerebral infarct due to thrombosis, normal pressure hydrocephalus |
| 4 | ≦65 yrs | Not carrier | MCI | MCI Aβ– | ≧27 | Q3 | Q3 | Q2 | Q3 | Q4 | No | possible burnout syndrome |
| 5 | >65 yrs | Carrier | MCI | MCI Aβ– | ≧27 | Q4 | Q4 | Q4 | Q4 | Q4 | No | - |

**Supplementary Table 1**

Details on false positives for the ROC analysis in the pre-PET MCI group for which the pooled variable obtained from LASSO was used to predict visual read positivity from amyloid PET

Q: Quartile of the whole dataset

**Supplementary Table 2**

Coefficients for contributions of different biomarkers to the pooled variable obtained with the LASSO regression in the pre-PET MCI group for predicting visual read positivity from amyloid PET

| **Variable** | **Logistic regression multivariable coeff.** | **LASSO coeff. unstandardized** |
| --- | --- | --- |
| Intercept | 1.635 | 0.7232 |
| Age | 0.064 | 0.0069 |
| Sex | 0.010 | 0.0004 |
| pGFAP | 0.291 | 0.0047 |
| pTau231 | 0.317 | 0.0408 |
| pTau181 | –0.076 | –0.0102 |
| pNfL | –0.094 | –0.0073 |
| pAβ42 | –0.253 | –0.1184 |
| pAβ42/40 | 0.128 | 10.4117 |
| pTau181/pAβ42 | –0.219 | –0.1728 |
